# Open thoracoabdominal aortic aneurysm repair in a patient with myasthenia gravis

**DOI:** 10.1093/icvts/ivab331

**Published:** 2021-11-24

**Authors:** Kyokun Uehara, Yasue Fujiwara, Manabu Morishima, Atsushi Iwakura

**Affiliations:** Department of Cardiovascular Surgery, Tenri Hospital, Nara, Japan

**Keywords:** Myasthenia gravis, Thoracoabdominal aortic aneurysm, Open repair, Graft replacement, Total intravenous anaesthesia

## Abstract

Myasthenia gravis (MG) is an autoimmune neuromuscular junction disorder and rarely coexists with aortic aneurysms requiring open repair. A 66-year-old patient with MG underwent extended thoraco-abdominal aortic aneurysm (TAAA) repair 16 years after onset of type-B acute aortic dissection. At 62 years, the patient was diagnosed with MG (MGFA class IIIa) from positive anti-acetylcholine receptor antibody without thymoma. Preoperatively, MG was well-controlled by prednisolone, cyclosporin and pyridostigmine. Extent II TAAA repair was performed under general anaesthesia maintained by total intravenous anaesthesia. Transcranial motor-evoked potential and somatosensory-evoked potential were applied to monitor intraoperative spinal cord ischaemia and muscle weakness. Amplitudes of motor-evoked potential and somatosensory-evoked potential attenuated intraoperatively but normalized after reperfusion from the reconstructed tube graft. Perioperative steroid coverage was given against surgical stress. The patient was weaned from mechanical ventilatory support on postoperative day 7. No signs of spinal cord ischaemia or muscle weakness were seen.

## CASE REPORT

**Figure 1:**
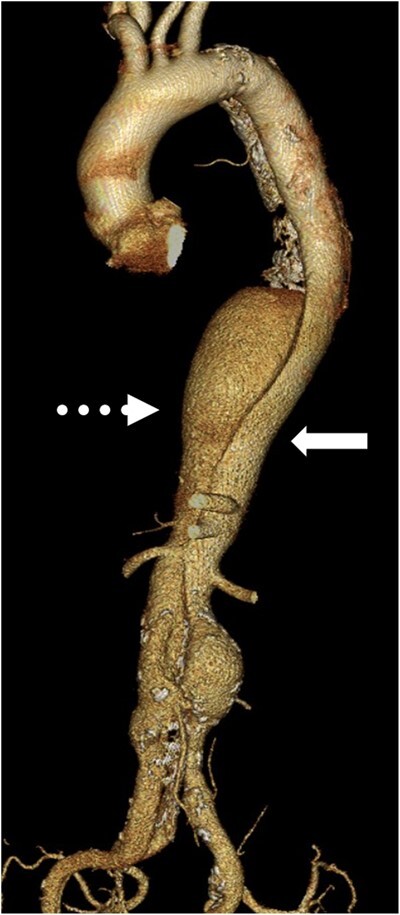
The preoperative computed tomography showing dissecting thoraco-abdominal aortic aneurysm with true lumen (solid arrow) and false lumen (dot arrow).

A 66-year-old patient with myasthenia gravis (MG) underwent extended thoraco-abdominal aortic aneurysm (TAAA) repair 16 years after onset of type-B acute aortic dissection. A pacemaker had been implanted due to sick sinus syndrome after catheter ablation for atrial fibrillation at 58 years. At 62 years, the patient was first diagnosed with MG (MGFA class IIIa) from a positive result for anti-acetylcholine receptor antibody. Thymoma was not detected on computed tomography. Immunosuppression therapy including prednisolone, pyridostigmine and cyclosporin following combination therapy with plasma exchanges and intravenous immunoglobulin resolved weakness of the ocular and skeletal muscles. The dissecting aneurysm gradually enlarged to >60 mm in size, so open extent II TAAA repair was planned. Preoperative computed tomography showed a partially thrombosed TAAA and the right renal artery arises from the false lumen (Fig. 1). The critical intercostal artery supplying the Adamkiewicz artery was located at the level of the left 10th intercostal space in the false lumen. Preoperative blood testing showed anti-acetylcholine receptor (anti-AChR) antibody level was 3.5 nmol/l, and MG was controlled by prednisolone at 5 mg/day, cyclosporin at 120 mg/day and pyridostigmine at 60 mg/day.

During surgery, general anaesthesia was maintained by total intravenous anaesthesia (TIVA) with target-controlled infusion propofol and remifentanil. Transcranial motor-evoked potential (MEP) and somatosensory-evoked potential (SEP) were applied to intraoperatively monitor spinal cord ischaemia and muscle weakness.

Mild permissive hypothermia (32°C) was administered, and then, the aorta was sequentially clamped and opened. A four-branched TAAA graft (J graft; Japan Lifeline, Tokyo, Japan) was anastomosed. The targeted intercostal arteries were reattached and immediately reperfused. During reconstruction, the amplitudes of both MEP and SEP attenuated but normalized after reperfusion from the reconstructed tube graft. The visceral arteries were also reconstructed separately.

**Figure 2:**
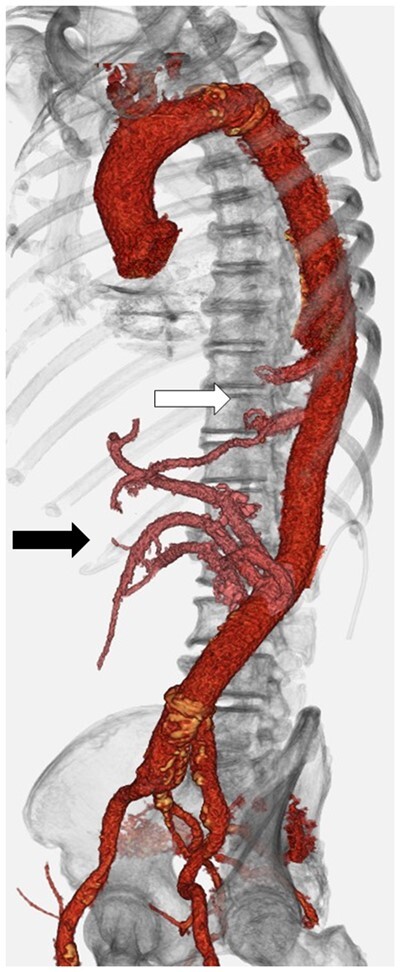
Reconstruction of intercostal artery—Adamkiewicz artery and the adjacent intercostal artery (white arrow). Visceral arteries were also replaced (black arrow).

After the patient was transferred to the intensive care unit, 50 mg of hydrocortisone was infused every 8 h, with the dose halved every day before starting tube feeding. The patient regained consciousness and spontaneous ventilation was established the next day. Blood testing on postoperative day 4 showed an anti-AChR antibody level of 1.2 nmol/l. The patient was weaned from mechanical ventilatory support on postoperative day 7. No signs of spinal cord ischaemia or muscle weakness were evident. Postoperative computed tomography showed intact reconstructed intercostal arteries and visceral arteries (Fig. 2).

## DISCUSSION

A small number of reports have described patients with MG undergoing cardiac surgeries, including coronary artery bypass grafting, valve repair or aortic arch replacement from a median sternotomy with or without thymectomy [1–4].

Various approaches to general anaesthesia have been reported for patients with MG [3–5]. A nondepolarizing neuromuscular blockade is generally selected to sustain appropriate relaxation. Reports indicate the benefit of sugammadex, which provides rapid and complete reversal of the neuromuscular blocking agents; therefore, anaesthetic management with rocuronium reversed by sugammadex has been widely applied even in patients with MG [5]. However, during TAAA repair, MEP monitoring has been preferred to prevent spinal cord ischaemia. We, therefore, believe that TIVA without neuromuscular blockade is a useful anaesthetic protocol during TAAA repair. This is the first case report to highlight extent II TAAA repair in a patient with MG using TIVA.

Hypothermia during cardiopulmonary bypass would reduce muscle strength [4]. Our patient showed moderate aortic valve insufficiency, and the distal arch was able to be clamped, so mild permissive hypothermia was administered to prevent spinal cord injury. Hybrid repair including total debranching bypass and stentgrafting or branched stentgrafting would be alternative procedures. In our case, the critical intercostal arteries and the right renal artery arise from the false lumen; therefore, the patient underwent open graft replacement.

One of the postoperative concerns in MG patients is respiratory failure [1–4]. Our patient required prolonged mechanical ventilatory support, but postoperative nerve conduction studies confirmed preservation of motor nerve conduction velocity and sensory nerve conduction velocity. The cause of prolonged mechanical ventilation would be the surgical invasiveness of the left thoracotomy, including differential lung ventilation and incision of the diaphragm. The amplitude of SEP did not attenuate after TAAA repair. Anti-AChR antibody concentration was 3.5 nmol/l before TAAA repair and 1.1 nmol/l on postoperative day 4. Decreases in anti-AChR antibody may be caused by haemodilution and adsorption due to the use of cardiopulmonary bypass during TAAA repair. Strict steroid coverage should be mandatory for MG patients after invasive surgeries, and our patient did not show any sign of muscle weakness or myasthenic crisis. Of course, good control of MG preoperatively would be the most important factor for TAAA repair.

**Conflict** **of interest:** none declared.

## Reviewer information

Interactive CardioVascular and Thoracic Surgery thanks Hakki Kazaz and the other, anonymous reviewer(s) for their contribution to the peer review process of this article.
